# Ten simple rules for choosing a PhD supervisor

**DOI:** 10.1371/journal.pcbi.1009330

**Published:** 2021-09-30

**Authors:** Loay Jabre, Catherine Bannon, J. Scott P. McCain, Yana Eglit

**Affiliations:** Department of Biology, Dalhousie University, Halifax, Nova Scotia, Canada; Dassault Systemes BIOVIA, UNITED STATES

## Introduction

The PhD beckons. You thought long and hard about why you want to do it, you understand the sacrifices and commitments it entails, and you have decided that it is the right thing for you. Congratulations! Undertaking a doctoral degree can be an extremely rewarding experience, greatly enhancing your personal, intellectual, and professional development. If you are still on the fence about whether or not you want to pursue a PhD, see [1,2] and others to help you decide.

As a PhD student in the making, you will have many important decisions to consider. Several of them will depend on your chosen discipline and research topic, the institution you want to attend, and even the country where you will undertake your degree. However, one of the earliest and most critical decisions you will need to make transcends most other decisions: choosing your PhD thesis supervisor. Your PhD supervisor will strongly influence the success and quality of your degree as well as your general well-being throughout the program. It is therefore vital to choose the right supervisor for you. A wrong choice or poor fit can be disastrous on both a personal and professional levels—something you obviously want to avoid. Unfortunately, however, most PhD students go through the process of choosing a supervisor only once and thus do not get the opportunity to learn from previous experiences. Additionally, many prospective PhD students do not have access to resources and proper guidance to rely on when making important academic decisions such as those involved in choosing a PhD supervisor.

In this short guide, we—a group of PhD students with varied backgrounds, research disciplines, and academic journeys—share our collective experiences with choosing our own PhD supervisors. We provide tips and advice to help prospective students in various disciplines, including computational biology, in their quest to find a suitable PhD supervisor. Despite procedural differences across countries, institutions, and programs, the following rules and discussions should remain helpful for guiding one’s approach to selecting their future PhD supervisor. These guidelines mostly address how to evaluate a potential PhD supervisor and do not include details on how you might find a supervisor. In brief, you can find a supervisor anywhere: seminars, a class you were taught, internet search of interesting research topics, departmental pages, etc. After reading about a group’s research and convincing yourself it seems interesting, get in touch! Make sure to craft an e-mail carefully, demonstrating you have thought about their research and what you might do in their group. After finding one or several supervisors of interest, we hope that the rules bellow will help you choose the right supervisor for you.

## Rule 1: Align research interests

You need to make sure that a prospective supervisor studies, or at the very least, has an interest in what you want to study. A good starting point would be to browse their personal and research group websites (though those are often outdated), their publication profile, and their students’ theses, if possible. Keep in mind that the publication process can be slow, so recent publications may not necessarily reflect current research in that group. Pay special attention to publications where the supervisor is senior author—in life sciences, their name would typically be last. This would help you construct a mental map of where the group interests are going, in addition to where they have been.

Be proactive about pursuing your research interests, but also flexible: Your dream research topic might not currently be conducted in a particular group, but perhaps the supervisor is open to exploring new ideas and research avenues with you. Check that the group or institution of interest has the facilities and resources appropriate for your research, and/or be prepared to establish collaborations to access those resources elsewhere. Make sure you like not only the research topic, but also the “grunt work” it requires, as a topic you find interesting may not be suitable for you in terms of day-to-day work. You can look at the “Methods” sections of published papers to get a sense for what this is like—for example, if you do not like resolving cryptic error messages, programming is probably not for you, and you might want to consider a wet lab–based project. Lastly, any research can be made interesting, and interests change. Perhaps your favorite topic today is difficult to work with now, and you might cut your teeth on a different project.

## Rule 2: Seek trusted sources

Discussing your plans with experienced and trustworthy people is a great way to learn more about the reputation of potential supervisors, their research group dynamics, and exciting projects in your field of interest. Your current supervisor, if you have one, could be aware of position openings that are compatible with your interests and time frame and is likely to know talented supervisors with good reputations in their fields. Professors you admire, reliable student advisors, and colleagues might also know your prospective supervisor on various professional or personal levels and could have additional insight about working with them. Listen carefully to what these trusted sources have to say, as they can provide a wealth of insider information (e.g., personality, reputation, interpersonal relationships, and supervisory styles) that might not be readily accessible to you.

## Rule 3: Expectations, expectations, expectations

A considerable portion of PhD students feel that their program does not meet original expectations [3]. To avoid being part of this group, we stress the importance of aligning your expectations with the supervisor’s expectations before joining a research group or PhD program. Also, remember that one person’s dream supervisor can be another’s worst nightmare and vice versa—it is about a good fit for you. Identifying what a “good fit” looks like requires a serious self-appraisal of your goals (see Rule 1), working style (see Rule 5), and what you expect in a mentor (see Rule 4). One way to conduct this self-appraisal is to work in a research lab to get experiences similar to a PhD student (if this is possible).

**Fig 1:**
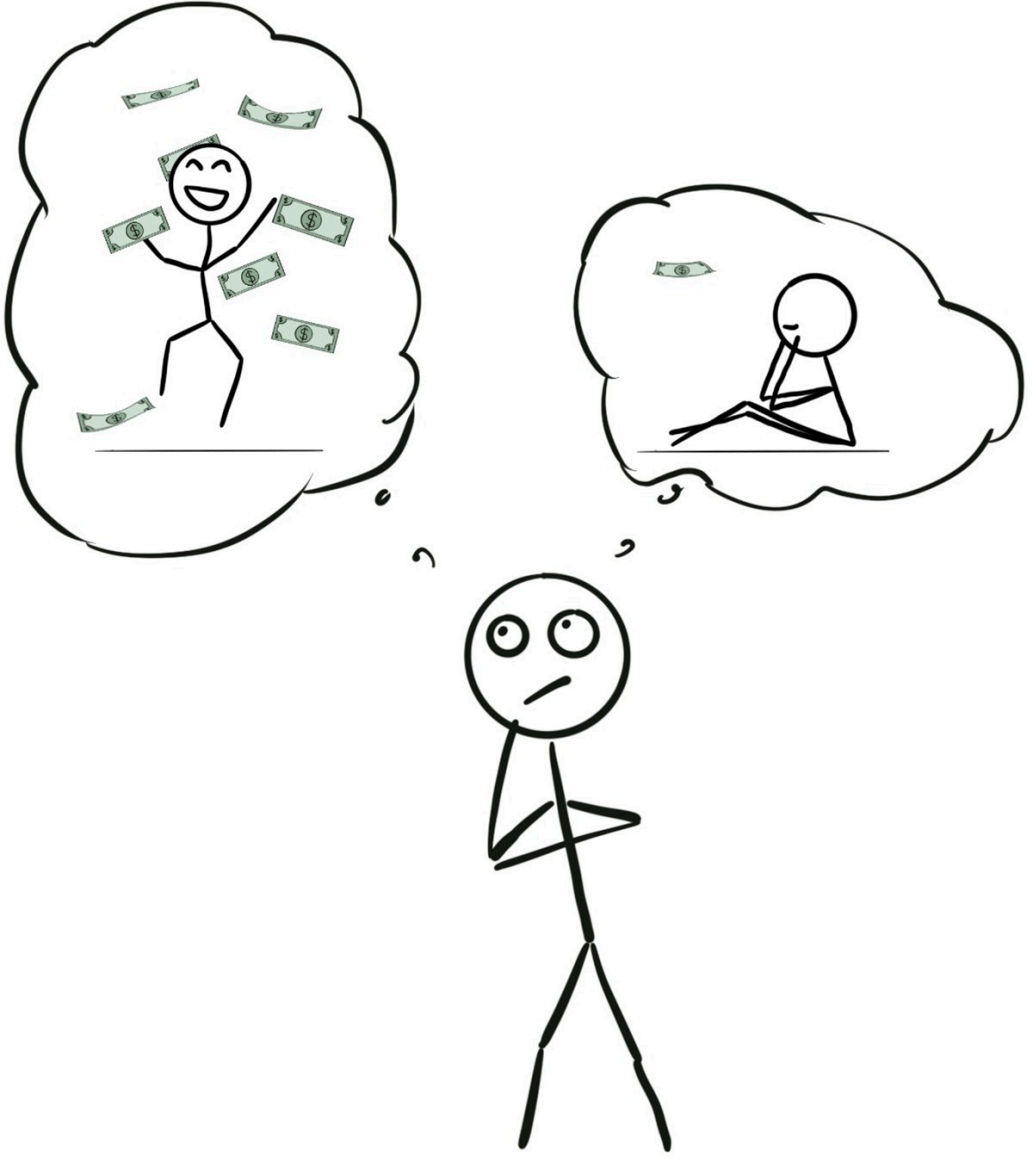
To avoid issues down the line, be sure to discuss personal and project finances with your potential supervisor.

Money!—Many people have been conditioned to avoid the subject of finances at all costs, but setting financial expectations early is crucial for maintaining your well-being inside and outside the lab. Inside the lab, funding will provide chemicals and equipment required for you to do cool research. It is also important to know if there will be sufficient funding for your potential projects to be completed. Outside the lab, you deserve to get paid a reasonable, livable stipend. What is the minimum required take-home stipend, or does that even exist at the institution you are interested in? Are there hard cutoffs for funding once your time runs out, or does the institution have support for students who take longer than anticipated? If the supervisor supplies the funding, do they end up cutting off students when funds run low, or do they have contingency plans? **(Fig 1).**

Professional development opportunities—A key aspect of graduate school training is professional development. In some research groups, it is normal for PhD students to mentor undergraduate students or take a semester to work in industry to get more diverse experiences. Other research groups have clear links with government entities, which is helpful for going into policy or government-based research. These opportunities (and others) are critical for your career and next steps. What are the career development opportunities and expectations of a potential supervisor? Is a potential supervisor happy to send students to workshops to learn new skills? Are they supportive of public outreach activities? If you are looking at joining a newer group, these sorts of questions will have to be part of the larger set of conversations about expectations. Ask: “What sort of professional development opportunities are there at the institution?”

Publications—Some PhD programs have minimum requirements for finishing a thesis (i.e., you must publish a certain number of papers prior to defending), while other programs leave it up to the student and supervisor to decide on this. A simple and important topic to discuss is: How many publications are expected from your PhD and when will you publish them? If you are keen to publish in high-impact journals, does your prospective supervisor share that aim? (Although question why you are so keen to do so, see the San Francisco Declaration on Research Assessment (www.sfdora.org) to learn about the pitfalls of journal impact factor.)

## Rule 4: It takes two to tango

Sooner or later, you will get to meet and interview with a prospective PhD supervisor. This should go both ways: Interview them just as much as they are interviewing you. Prepare questions and pay close attention to how they respond. For example, ask them about their “lab culture,” research interests (especially for the future/long term), and what they are looking for in a graduate student. Do you feel like you need to “put on an act” to go along with the supervisor (beyond just the standard interview mode)? Represent yourself, and not the person you think they are looking for. All of us will have some interviews go badly. Remember that discovering a poor fit during the interview has way fewer consequences than the incompatibility that could arise once you have committed to a position.

To come up with good questions for the prospective supervisor, first ask yourself questions. What are you looking for in a mentor? People differ in their optimal levels of supervision, and there is nothing wrong with wanting more or less than your peers. How much career guidance do you expect and does the potential supervisor respect your interests, particularly if your long-term goals do not include academia? What kind of student might not thrive in this research group?

Treat the PhD position like a partnership: What do you seek to get out of it? Keep in mind that a large portion of research is conducted by PhD students [4], so you are also an asset. Your supervisor will provide guidance, but the PhD is your work. Make sure you and your mentor are on the same page before committing to what is fundamentally a professional contract akin to an apprenticeship (see “Rule 3”).

## Rule 5: Workstyle compatibility

Sharing interests with a supervisor does not necessarily guarantee you would work well together, and just because you enjoyed a course by a certain professor does not mean they are the right PhD supervisor for you. Make sure your expectations for work and work–life approaches are compatible. Do you thrive on structure, or do you need freedom to proceed at your own pace? Do they expect you to be in the lab from 6:00 AM to midnight on a regular basis (red flag!)? Are they comfortable with you working from home when you can? Are they around the lab enough for it to work for you? Are they supportive of alternative work hours if you have other obligations (e.g., childcare, other employment, extracurriculars)? How is the group itself organized? Is there a lab manager or are the logistics shared (fairly?) between the group members? Discuss this before you commit!

Two key attributes of a research group are the supervisor’s career stage and number of people in the group. A supervisor in a later career stage may have more established research connections and protocols. An earlier career stage supervisor comes with more opportunities to shape the research direction of the lab, but less access to academic political power and less certainty in what their supervision style will be (even to themselves). Joining new research groups provides a great opportunity to learn how to build a lab if you are considering that career path but may take away time and energy from your thesis project. Similarly, be aware of pros and cons of different lab sizes. While big labs provide more opportunity for collaborations and learning from fellow lab members, their supervisors generally have less time available for each trainee. Smaller labs tend to have better access to the supervisor but may be more isolating [5,6]. Also note that large research groups tend to be better for developing extant research topics further, while small groups can conduct more disruptive research [7].

## Rule 6: Be sure to meet current students

Meeting with current students is one of the most important steps prior to joining a lab. Current students will give you the most direct and complete sense of what working with a certain supervisor is actually like. They can also give you a valuable sense of departmental culture and nonacademic life. You could also ask to meet with other students in the department to get a broader sense of the latter. However, if current students are not happy with their current supervisor, they are unlikely to tell you directly. Try to ask specific questions: “How often do you meet with your supervisor?”, “What are the typical turnaround times for a paper draft?”, “How would you describe the lab culture?”, “How does your supervisor react to mistakes or unexpected results?”, “How does your supervisor react to interruptions to research from, e.g., personal life?”, and yes, even “What would you say is the biggest weakness of your supervisor?”

## Rule 7: But also try to meet past students

While not always possible, meeting with past students can be very informative. Past students give you information on career outcomes (i.e., what are they doing now?) and can provide insight into what the lab was like when they were in it. Previous students will provide a unique perspective because they have gone through the entire process, from start to finish—and, in some cases, no longer feel obligated to speak well of their now former supervisor. It can also be helpful to look at previous students’ experiences by reading the acknowledgement section in their theses.

## Rule 8: Consider the entire experience

Your PhD supervisor is only one—albeit large—piece of your PhD puzzle. It is therefore essential to consider your PhD experience as whole when deciding on a supervisor. One important aspect to contemplate is your mental health. Graduate students have disproportionately higher rates of depression and anxiety compared to the general population [8], so your mental health will be tested greatly throughout your PhD experience. We suggest taking the time to reflect on what factors would enable you to do your best work while maintaining a healthy work–life balance. Does your happiness depend on surfing regularly? Check out coastal areas. Do you despise being cold? Consider being closer to the equator. Do you have a deep-rooted phobia of koalas? Maybe avoid Australia. Consider these potentially even more important questions like: Do you want to be close to your friends and family? Will there be adequate childcare support? Are you comfortable with studying abroad? How does the potential university treat international or underrepresented students? When thinking about your next steps, keep in mind that although obtaining your PhD will come with many challenges, you will be at your most productive when you are well rested, financially stable, nourished, and enjoying your experience.

## Rule 9: Trust your gut

You have made it to our most “hand-wavy” rule! As academics, we understand the desire for quantifiable data and some sort of statistic to make logical decisions. If this is more your style, consider every interaction with a prospective supervisor, from the first e-mail onwards, as a piece of data.

**Fig 2:**
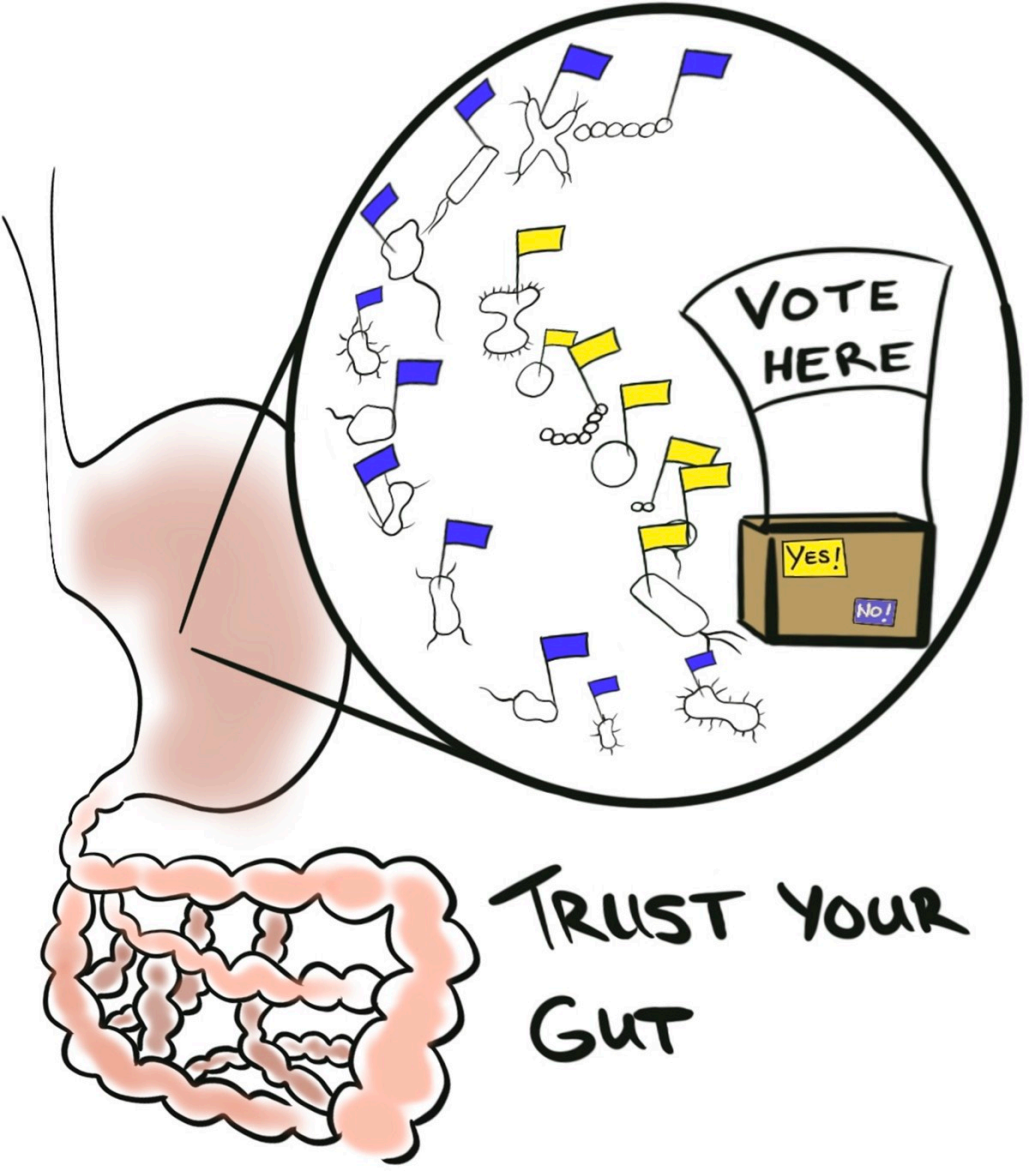
Be conscious of “alarm bells” and “good gut feelings” as you think about a potential supervisor—believe in the power of instinct.

However, there is considerable value in trusting gut instincts. One way to trust your gut is to listen to your internal dialogue while making your decision on a PhD supervisor. For example, if your internal dialogue includes such phrases as “it will be different for me,” “I’ll just put my head down and work hard,” or “maybe their students were exaggerating,” you might want to proceed with caution. If you are saying “Wow! How are they so kind and intelligent?” or “I cannot wait to start!”, then you might have found a winner **(Fig 2).**

## Rule 10: Wash, rinse, repeat

**Fig 3:**
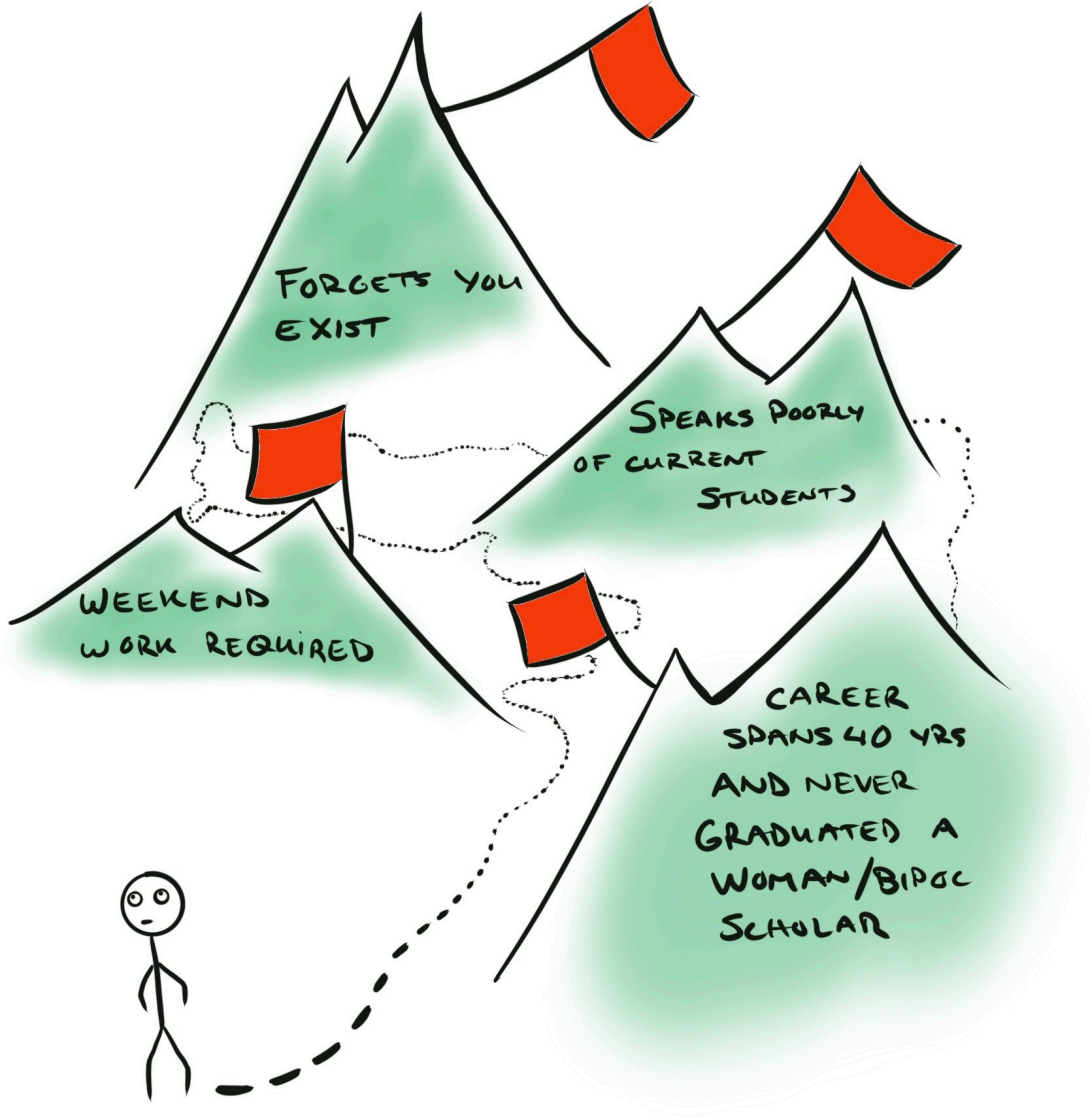
Watch out for red flags as you evaluate potential supervisors. The more supervisors your interview and the more advice you get from peers, the more apparent these red flags will become.

The last piece of advice we give you is to do this lengthy process all over again. Comparing your options is a key step during the search for a PhD supervisor. By screening multiple different groups, you ultimately learn more about what red flags to look for, compatible work styles, your personal expectations, and group atmospheres. Repeat this entire process with another supervisor, another university, or even another country. We suggest you reject the notion that you would be “wasting someone’s time.” You deserve to take your time and inform yourself to choose a PhD supervisor wisely. The time and energy invested in a “failed” supervisor search would still be far less than what is consumed by a bad PhD experience **(Fig 3).**

## Conclusions

Pursuing a PhD can be an extremely rewarding endeavor and a time of immense personal growth. The relationship you have with your PhD supervisor can make or break an entire experience, so make this choice carefully. Above, we have outlined some key points to think about while making this decision. Clarifying your own expectations is a particularly important step, as conflicts can arise when there are expectation mismatches. In outlining these topics, we hope to share pieces of advice that sometimes require “insider” knowledge and experience.

After thoroughly evaluating your options, go ahead and tackle the PhD! In our own experiences, carefully choosing a supervisor has led to relationships that morph from mentor to mentee into a collaborative partnership where we can pose new questions and construct novel approaches to answer them. Science is hard enough by itself. If you choose your supervisor well and end up developing a positive relationship with them and their group, you will be better suited for sound and enjoyable science.
